# The Beneficial Effects of Nordic Walking Training Combined with Time-Restricted Eating 14/24 in Women with Abnormal Body Composition Depend on the Application Period

**DOI:** 10.3390/nu16101413

**Published:** 2024-05-08

**Authors:** Olga Czerwińska-Ledwig, Joanna Kryst, Ewa Ziemann, Andżelika Borkowska, Joanna Reczkowicz, Adrianna Dzidek, Łukasz Rydzik, Tomasz Pałka, Małgorzata Żychowska, Wojciech Kupczak, Marta Mydlárová Blaščáková, Anna Piotrowska

**Affiliations:** 1 Institute for Basic Sciences, Faculty of Physiotherapy, University of Physical Education, 31-571 Krakow, Poland; olga.czerwinska@awf.krakow.pl (O.C.-L.); joanna.kryst@awf.krakow.pl (J.K.); adrianna.dzidek@doctoral.awf.krakow.pl (A.D.); 2 Department of Athletics, Strength and Conditioning, Poznan University of Physical Education, 61-871 Poznan, Poland; ziemann@awf.poznan.pl; 3 Department of Bioenergetics and Physiology of Exercise, Medical University of Gdansk, 80-210 Gdansk, Poland; andzelika.borkowska@gumed.edu.pl (A.B.); joanna.reczkowicz@gumed.edu.pl (J.R.); 4 Institute of Sports Sciences, University of Physical Education, 31-571 Krakow, Poland; lukasz.rydzik@awf.krakow.pl; 5 Department of Physiology and Biochemistry, Faculty of Physical Education and Sport, University of Physical Education, 31-571 Krakow, Poland; tomasz.palka@awf.krakow.pl; 6 Faculty of Health Sciences and Physical Culture, Biological Fundation of Physical Culture, Kazimierz Wielki 10 University in Bydgoszcz, 85-064 Bydgoszcz, Poland; zychowska.m@gmail.com; 7 Student’s Science Club, Department of Chemistry and Biochemistry, University of Physical Education, 31-571 Krakow, Poland; 8 Department of Biology, Faculty of Humanities and Natural Sciences, University of Presov, 08-116 Presov, Slovakia; marta.blascakova@unipo.sk

**Keywords:** leptin, lipid profile, Nordic walking, resistin, time-restricted eating

## Abstract

The aim of the study was to assess the impact of two lengths of Nordic walking (NW) training interventions combined with time-restricted eating (TRE) on improving body-composition parameters, lipid profiles, and levels of selected adipokines in women with elevated body mass. Overweight and obese women (*n* = 55, age: 21–85) were recruited. Four groups were selected: 6 weeks (SG6, *n* = 13) and 12 weeks intervention (SG12, *n* = 13); and two control groups: CON6 (*n* = 13) and CON12 (*n* = 13). The training sessions took place three times a week (60 min each) and were conducted outdoors under the supervision of a professional coach. The training intensity was determined individually. The extended NW program combined with TRE induced a significant weight reduction in SG12 by 1.96 kg (*p* = 0.010) and fat tissue by 1.64 kg (*p* = 0.05). The proposed interventions did not affect LBM, TBW [kg], VFA, and lipid profile. The LDL/HDL ratio changed with a small size effect. The leptin concentration differed between groups (*p* = 0.006), but not over time. For resistin, the differentiating factor was time (*p* = 0.019), with lower results observed after the intervention. The change in leptin concentration was negatively correlated with its baseline concentration (*p* = 0.025). Extended to 12 weeks, this intervention allows for an improvement in body composition. Neither 6 nor 12 weeks of training and fasting affected the lipoprotein profile. It is, therefore, indicated to recommend prolonged training protocols and to inform patients that beneficial effects will be seen only after prolonged use of training and time-restricted eating.

## 1. Introduction

Obesity, which is associated with an excessive accumulation or improper distribution of adipose tissue, exerts a significant impact on health. Body adiposity is considered to be an atherogenic factor, correlating with the occurrence of insulin and leptin resistance and aberrant lipid metabolism, thereby leading to the onset of numerous civilization-related diseases and increasing the risk of mortality [1]. According to the World Health Organization, in 2016, overweight affected 1.9 billion individuals (39%), while obesity affected 650 million adults (13%) globally, indicating the emergence of an obesity pandemic [1].

Individuals with obesity are recommended to modify their lifestyle, primarily by increasing physical activity while simultaneously creating an energy deficit through dietary intake, resulting in gradual weight reduction [2]. One of the recommended types of physical activity for this group, regardless of age, is Nordic walking (NW) training [3,4]. Due to the engagement of both upper and lower body muscles, the energy cost of this activity is higher than that of regular walking [5], with a simultaneous reduction in load on the knee and intervertebral joints [6]. This type of training may be recommended for patients with cardiovascular diseases [7], oncological conditions [8,9], or neurological disorders [10,11,12]. Such a wide range underscores the safety of this training modality. The effectiveness of various forms of NW training has been investigated so far. This form of physical activity could also be combined with other procedures, such as cold exposure or time-restricted eating [13,14].

Time-restricted eating (TRE), a temporal pattern of food-intake delivery, is increasingly utilized for health purposes, including weight reduction [15,16,17]. Additionally, TRE enhances the body’s ability to defend against oxidative and metabolic stress [18]. The human body’s adaptation to fasting is crucial for survival and well-being during periods of abstinence, and its implementation provides numerous health benefits, particularly for individuals with excessive adipose-tissue content. Under the influence of fasting, the body, instead of depending entirely on glucose stored in liver glycogen, also utilizes energy from ketones derived from adipose tissue [19]. The increase in blood ketone levels may initiate the activation of various intracellular signaling pathways, which, among other functions, slow down the aging process [20] and regulate the expression and activity of numerous proteins, including sirtuins [20,21,22].

Increased white-adipose-tissue (WAT) content, which constitutes the hallmark of obesity, leads to enhanced secretion of adipokines, including leptin, resistin, interleukin 6 (IL-6), tumor necrosis factor-alpha (TNF-α), visfatin, vaspin, and retinol-binding protein 4 (RBP-4). 

Leptin is an adipokine involved in both the regulation of body weight and the regulation of hunger and appetite [23]. Elevated leptin levels are observed in obese individuals [24], which decrease with weight loss, adjusting the body’s metabolism to the energy reserves stored in adipose tissue [25]. Decreased leptin levels have been observed under the influence of starvation, as well as after following a low-calorie diet [26] and after physical training [27].

In obesity, an increase in the serum resistin concentration is observed [28]. Its action is associated with the development of insulin resistance, and the inhibition of resistin activity lowers serum glucose levels and increases insulin sensitivity [29], which has favorable clinical significance. There is scientific evidence of the impact of various forms of physical activity on a significant reduction in resistin levels in obese individuals [30,31].

Women represent a group with significantly greater constraints stemming from anxiety about engaging in physical activity [32]. Above the age of 35, a considerable decrease in muscle mass (especially in the legs), along with an increase in the percentage of body fat, occurs [33]. Generally, women tend to have higher body mass index (BMI) values than men [34]. There are also intensified issues with maintaining a proper diet, often attributed to the phenomenon of emotional eating and stress relief [35]. The abovementioned considerations indicate that overweight and obesity pose a significant challenge for individuals of this gender. In line with the above, given the high acceptability of physical activity in the form of NW training [36], this study was undertaken. Its main aim was to assess the impact of two lengths of NW training interventions combined with TRE on improving body-composition parameters, lipid profiles, and levels of selected adipokines in women with an abnormal body composition. It was hypothesized that a 6-week intervention involving a combination of two stimuli, namely training and dietary, would result in significant changes, including a reduction in body weight associated with a loss of body fat and fat tissue mass; improvement in the serum lipid profile; and reduction in the levels of selected adipokines. Additionally, it was chosen to compare the 6-week intervention with a 12-week one.

## 2. Materials and Methods

### 2.1. Study Group

Overweight and obese women with BMIs over 25 [2] and no physical activity other than housework were invited to participate in the project. Medical qualification included verification of contraindications to physical exertion (walking with poles) and the presence of glycemic disorders allowing for the diagnosis of diabetes. Exclusion criteria from the study were dietary changes within 3 months prior to participating in the research project, health problems of neurological or orthopedic origin, participation in other physical activities during the project, and the use of supplementation or medications affecting lipid and carbohydrate metabolism.

The project obtained approval from the Ethics Committee (58/KBL/OLI/2022 dated 11 April 2022). Participants were informed about the purpose and research methods and provided written consent to participate in the study. They were also informed about the possibility of withdrawing from participation at any study stage without providing a reason. Each participant also had the opportunity to access their individual results.

**Figure 1:**
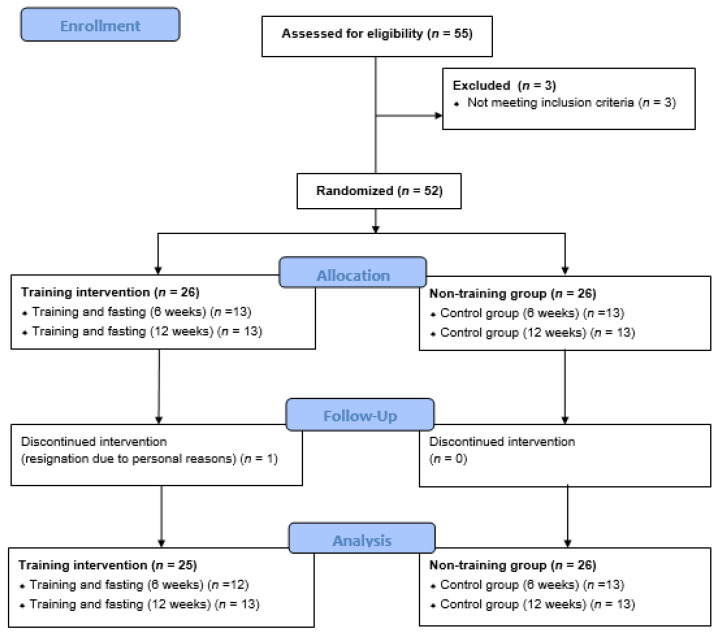
CONSORT patient flow diagram.

Four groups were selected through randomization. The two undergoing interventions were women participating in training sessions and 14 h fasting for 6 (SG6, *n* = 13) and 12 weeks (SG12, *n* = 13); and the two control groups were CON6, *n* = 13; and CON12, *n* = 13. Initially, the groups were formed consisting of individuals who indicated their ability to participate for either 6 or 12 weeks. Subsequently, within this subset of participants committed to the designated timeframe, randomization was conducted to assign individuals to either the intervention or control group (drawing of black and white balls). The patient flow diagram is presented in Figure 1.

### 2.2. Study Protocol

The study on the design of a randomized controlled trial was interventional. Each participant underwent an initial examination by a doctor to assess the presence of the inclusion and exclusion criteria for the project. Subsequently, randomization was conducted to allocate participants to groups. Individual training loads were then determined for the active groups, while the groups practicing fasting were instructed on the principles of time-restricted eating. NW training sessions lasted for either 6 weeks or 12 weeks (3 sessions per week).

In all groups, body-composition analysis was performed twice (before and after the completion of the training series, in control groups, and at the same time points). Blood samples were collected before the first training session, as well as before the last training session. In the control groups, blood was drawn twice at the corresponding time points.

### 2.3. Time-Restricted Eating

Fasting was conducted daily (7 days a week) for 14 h per day within individually chosen period intervals by the participants. The feeding window lasted for 10 h. In addition to this modification, participants were instructed not to change their dietary habits and to continue their self-composed diet as before the project commenced. Adherence to time-restricted eating was monitored by the trainer and other researchers when speaking to the participants during each training session.

### 2.4. NW Training

The NW training program was developed by a qualified trainer based on the available literature [37]. The training sessions took place three times a week (Monday, Wednesday, and Friday) and lasted 60 min each. The sessions were conducted under the supervision of the trainer outdoors in green areas. Before the first session, each participant had the length of the poles adjusted individually. The trainer and other researchers were controlling the walking technique with the poles during the training sessions. 

The physical performance of the participants was determined using the 2 km walking test (UKK Walk Test) [38] with the following formula:

UKK = 304 − [(8.5Tmin + 0.14Ts + 0.32HR + 1.1BMI) − 0.4A]

where Tmin is total minutes of walking; Ts is seconds of walking in the last incomplete minute; HR is heart rate; BMI is body mass index; and A is age in years.

Then, the VO_2_max level was determined individually for each participant.

VO_2_max = 116.2 − 2.98T − 0.11HR − 0.14A − 0.39BMI

where: A is age in years; BMI is body mass index; HR is heart rate; and T is walking time in minutes converted to the decimal system.

HRmax was calculated for each participant individually with the use of the Nes formula [39], which is suitable for subjects in different age groups:

HRmax = 211 − 0.64 age

Based on this, a threshold of 70% of HRmax was determined, which individual subjects should not exceed during training sessions to maintain a moderate exercise intensity.

The training consisted of 3 stages. The first stage involved familiarizing the participants with the correct Nordic walking technique. The second stage included strength-resistance and stretching exercises to improve motor skills, flexibility, and joint range of motion, and aerobic exercises preparing for more intense efforts. The third stage involved conditioning training to improve endurance capabilities, primarily based on walking with poles for a specified duration (aerobic training and interval training).

The structure of each training unit (60 min) included a warm-up of 10–15 min; the main part of 40–45 min with assumed training intensity; and a cooling down part of 5–15 min (stretching exercises and breathing exercises). Training intensity was monitored using sports testers (M400 Polar, Kempele, Finland) with individually entered user data, including acceptable heart rate levels. If these were exceeded, the device beeped, and the instructor adjusted the patient’s exercise intensity. 

### 2.5. Somatic Measurements and Assessment of Body Composition

Participants were subjected to measurements of body mass (BM) and body composition before and after the training series, as well as a one-time measurement of body height (BH). The indices for analyzing body composition were determined using the Jawon Medical IOI-353 body-composition analyzer (certified EC0197, Jawon Medical, Yuseong, Republic of Korea). The following variables were estimated: lean body mass (LBM), soft lean mass (SLM), percent total body water (%TBW), total body water (TBW), percent body fat (%PF), fat mass (FM), and visceral fat area (VFA). Body height (BH) was measured using a Martin-type anthropometer (GPM, Rudolf Martin, LLC, USA) with a measurement accuracy of 0.5 cm. Based on these parameters, the BMI was calculated.

### 2.6. Blood-Parameters Measurements

Pre-training blood samples were collected after an overnight fast, followed by standardized breakfast consumption, before the participants engaged in training. Post-training blood collection took place immediately after exercise cessation, no later than 15 min thereafter.

In accordance with prevailing standards, a laboratory diagnostician collected venous blood from the elbow bend into Vacumed^®^ vacuum system tubes (F.L. Medical, Torreglia, Italy). Blood was collected into 3 tubes—1 containing EDTA K2 (dipotassium salt of ethylenediamine tetraacetic acid) and 2 containing coagulation activator. Tubes with a coagulation activator were left to clot for 15 min, then centrifuged (10 min; 5°C).

The blood samples collected in EDTA and 1 sample with a coagulation activator were sent for analysis to a medical diagnostic laboratory following a generally accepted methodology. The blood collected into the EDTA tubes was used for determining morphological blood parameters. Serum obtained from blood samples collected on the clot was used for lipid-profile determinations (total cholesterol concentration [TC], high-density lipoprotein cholesterol concentration [HDL-C], triglyceride concentration [TG]), and glucose.

Based on these parameters, subsequent indices were calculated, including the atherogenic index (AIP) [40] and the LDL/HDL ratio, as predictors of cardiovascular diseases.

AIP = log(TG/HDL-C)

The material from the last of the tubes containing the coagulation activator was centrifuged for 10 min at 4 °C and 2500 rpm, and the resulting supernatant, showing no signs of hemolysis, was transferred to Eppendorf-type microtubes. In this form, they were frozen in a low-temperature freezer (−80 °C) until biochemical assays were performed. The determination of the leptin and resistin concentrations in serum was carried out using an immunoenzymatic method (ELISA test) utilizing the multiplex method (Magpix Luminex, Xmap instrument; Luminex Corporation, Austin, TX, USA).

### 2.7. Statistical Analysis

All statistical analyses were conducted using JASP 0.16.4 software (University of Amsterdam, Amsterdam, The Netherlands). The obtained results were presented using descriptive statistics (mean ± standard deviation, SD). For biochemical variables, as well as body-composition parameters, a repeated measures ANOVA (RMANOVA) analysis was performed, preceded by an assessment of assumptions (normality, variance, and sphericity). If the sphericity assumption was violated (Mauchly’s test), Greenhouse–Geisser or Huynh–Feldt corrections were applied. In the case of non-normal distribution assumption (Shapiro–Wilk test) and/or heterogeneity of variances (Levene’s test), the non-parametric Friedman test was used. Statistically significant RMANOVA results for group, time, and group × time interaction parameters were subjected to post hoc Holm–Bonferroni tests. The effect size was assessed using the Eta squared coefficient (η^2^) and interpreted as follows: 0.1—small; 0.25—medium; 0.37–large size effect.

A correlation analysis was also performed, depending on the normality of the variable’s distribution, using the Pearson correlation coefficient or Spearman’s rho.

## 3. Results

The results of the body-composition measurements are presented in Table 1. Significant differences were indicated for BM (*p* = 0.018). The differences were observed between measurements before and after the training series in the SG12 group (*p* = 0.010), where the mean weight loss was 1.96 (±1.59) kg. For the group training for 6 weeks, the difference was 1.59 (±1.11) kg but did not reach statistical significance (*p* = 0.081). Both control groups did not significantly change their body weight. The BM changes in the groups were as follows. In the SG12 group, a decrease on average by 1.96 ± 1.66 kg was observed. In the SG6 group, BM decreased by 1.59 ± 1.16 kg. In the CON 12 group, there was a decrease of 0.13 ± 2.42, and in CON6, an increase of 0.75 ± 1.29 kg. Statistically significant differences were observed between the groups CON6 vs. SG6 (*p* = 0.006) and CON6 vs. SG12 (*p* = 0.001). A trend close to statistical significance was observed between the SG12 and CON12 groups (*p* = 0.057). The proposed interventions did not affect LBM, TBW [kg], and VFA.

For the variable BF [kg], the differentiating factor was time (*p* = 0.021). Differences for the parameters group and interaction (time × group) were within the range of statistical trend (*p* = 0.074 and 0.060, respectively). Further analyses indicated lower results for the SG12 group after the training series (*p* = 0.050, a value on the borderline between a significant difference and within the range of a statistical trend). The mean fat tissue loss was 1.64 kg. Assessing the variable BFP showed significance for the time parameter (<0.001), while the group parameter and interaction were insignificant. Post hoc tests revealed significant differences for the SG12 group after the training series compared to baseline and compared to the control (mean BFP loss: 1.44%). During this analysis, it was also revealed that the CON12 group significantly increased the percentage of body fat content over 12 weeks.

Statistically significant differences were indicated for TBW [%], and these differences pertained to pre- and post-intervention results for the CON12 group (*p* = 0.017), SG6 group (*p* = 0.005), and SG12 group (*p* < 0.001). In all indicated groups, an increase in TBW was observed. BMI significantly differed for the group x time interaction (*p* = 0.012). Significant results were shown for the SG12 group post-intervention compared to the control group.

The results of the biochemical tests are presented in Table 2. A statistical analysis indicated that the proposed interventions did not affect the levels of TC, LDL, and TG in the serum of the examined women. For HDL, significance in terms of statistical trend (*p* = 0.057) for the combined effect of factors: time and group can be indicated. Significant differences were obtained for the LDL/HDL ratio, where the results before the project differed significantly from the results obtained after the intervention (*p* = 0.011, η^2^ = 0.023). An interaction between time and group was also indicated (*p* = 0.048, η^2^ = 0.034). However, post hoc tests did not reveal significant differences in comparisons between the two groups. The calculated AIP index did not differentiate the examined groups at the assumed significance level. The conducted analysis also did not show significant differences in blood glucose levels.

Another group of examined parameters is blood morphological markers. The results obtained in the project are presented in Table 3. Significant differences were indicated for the quantity of leukocytes, erythrocytes, blood platelets, percentage of neutrophils, and monocytes. Significant differences were also noted in the results of HCT in relation to time—with lower values after interventions but a low effect size (*p* = 0.042, η^2^ = 0.012).

For the leukocyte count, the differentiating factors were time (*p* = 0.030, η^2^ = 0.009) and group (*p* = 0.017, η^2^ = 0.209). Differences were noted in measurements before and after the training series (*p* = 0.030), and the post hoc test indicated differences between the SG6 group and the CON6 group (*p* = 0.039).

The erythrocyte count was differentiated by time, but the effect size was small (*p* = 0.038, η^2^ = 0.007). Differences were also noted in measurements before and after the training series (*p* = 0.030). However, post hoc tests did not reveal significant differences. Characteristics of the erythrocytes also differed. For the variable MCV (η^2^ = 0.253), significant differences were indicated between SG12 and the control (*p* = 0.050; value on the borderline between a significant difference and within the range of a statistical trend), as well as between SG6 and SG12 (*p* = 0.038). The examined groups also differed regarding the MCHC variable (η^2^ = 0.187). Significant differences were detected between SG12 and its corresponding control group (*p* = 0.017). A difference was indicated between the two intervention groups in terms of statistical trend (*p* = 0.065). For the variable HCT, the differentiating factor was time (*p* = 0.042, η^2^ = 0.012). 

The platelet count differed between groups (*p* = 0.037, η^2^ = 0.179). However, in seeking groups that elicited this effect, only a trend between SG6 and SG12 was noted (*p* = 0.088). Differences were also found for the percentages of neutrophils (time: *p* = 0.043, η^2^ = 0.010; group: *p* = 0.043, η^2^ = 0.166) where the values measured after intervention were higher in the SG6 and CON6 groups. However, this was not confirmed in the post hoc analysis. For monocytes, significant differences were also found (time: *p* = 0.038, η^2^ = 0.010; group: *p* = 0.004, η^2^ = 0.253). Monocyte counts were higher in the second measurement in all groups except CON12. Statistically significant differences were found between groups SG6 (in which the counts were highest) and CON 12 (*p* = 0.011), as well as between SG6 and SG12 (in which the counts were lowest) (*p* = 0.009) This result was primarily driven by the difference between the SG6 group and the control group. Percentages of lymphocytes, eosinophils, and basophils did not differ between the examined groups.

**Figure 2:**
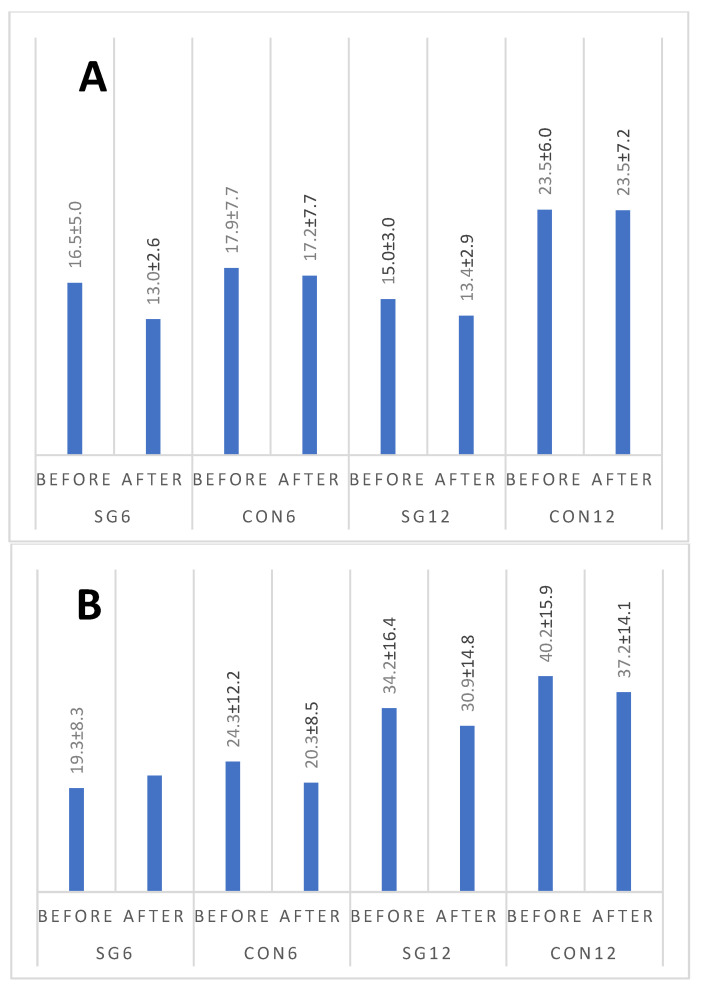
Changes in resistin (**A**, pg/mL) and leptin (**B**, ng/mL) levels in female subjects. SG6 group subjected to training intervention and time-restricted eating for 6 weeks; CON6 control group observed for 6 weeks; SG12 group subjected to training intervention and time-restricted eating for 12 weeks; CON12 control group observed for 12 weeks.

Figure 2A,B presents changes in leptin and resistin concentrations. A statistical analysis indicated that the leptin concentration differed between groups (*p* = 0.006). However, the results for neither group changed over time. For resistin, the differentiating factors were time (*p* = 0.019, with lower results observed after the intervention) and group (*p* = 0.001, with higher results observed for the CON12 group). No significant interaction was found (*p* = 0.231).

In searching for correlations between the examined adipokines and other variables, significant associations were identified only for leptin. An inverse correlation was demonstrated between leptin and SLM (r = 0.424, *p* = 0.006), as well as direct proportional relationships with the percentage of body fat (r = 0.52, *p* < 0.001), visceral fat area (VFA) (r = 0.446, *p* = 0.004), and body mass (r = 0.363, *p* = 0.021). Moreover, the magnitude of change in the leptin concentration obtained after the interventions was found to be dependent on SLM (r = 0.319, *p* = 0.045). The results of significant correlations between the examined adipokines and body composition are presented in Table 4.

## 4. Discussion

The results presented in this study indicate the effectiveness of the intervention in the form of NW training combined with TRE (14/24) only in certain areas examined. Beneficial effects were observed with the extended intervention (12 weeks), primarily concerning improvements in body composition and changes in blood morphology. However, neither the lipid-profile results nor the calculated indices (AIP and LDL/HDL ratio) demonstrated improvement. There was also no significant change in fasting glucose levels in response to both times of the time-restricted eating duration. On the other hand, Kortas et al. recently observed that NW supported by IF ameliorated glycated hemoglobin [13].

Both NW training and modified, low-calorie dietary intervention were shown to contribute to weight loss and improvement in lipid profiles in obese men [41]. NW training of varying duration and intensity tailored to the fitness levels of participants leads to improvements in body composition, reduction in waist circumference, improvement in lipid profile and adipokines, carbohydrate metabolism, reduction in oxidative stress, and inflammatory state in women with various degrees of obesity [42,43,44,45,46]. Physical activity, through increased cellular metabolism affecting adipose tissue, indirectly influences its metabolites.

It is indicated that NW training, used as an intervention to modify body composition, demonstrates greater effectiveness when supervised and conducted by qualified trainers [47]. Therefore, in this project, supervised NW training conducted by a qualified trainer was implemented. The training sessions were conducted on soft surfaces to allow for full utilization of the NW technique and to protect the joints of the participants. This is particularly important for individuals with obesity, where joint overload may be more pronounced. The same group of authors cited earlier also pointed out the greater effectiveness of NW compared to walking training without poles [47]. NW training is also highly popular among various groups of patients requiring rehabilitation-focused training interventions, as evidenced by high adherence to training regimens, which was also observed in this project.

Analyzing the impact of the proposed intervention on body composition, a beneficial effect of combined training with TRE conducted over 12 weeks was observed on body mass (however, we observed a small effect size), as well as a reduction in fat tissue content (both expressed in kg—small effect size; and as a percentage of total body mass, large effect size). These data are highly significant for the selected group, which is characterized by abnormal body composition indicative of overweight or obesity. For CON12, it was indicated that after 12 weeks of observation, BFP was significantly higher than before starting the project, which is a characteristic manifestation of the progression of obesity as a disease. Similar effects have been observed earlier [48], indicating the necessity of introducing interventions for obese individuals, as leaving them without any actions leads to disease progression.

On the other hand, it is not surprising that the combination of fasting with NW showed favorable effects on body composition, considering that beneficial effects on body mass and BMI had already been observed for isolated interventions in the form of intermittent fasting [49]. However, in these studies, fasting encompassed 10 h, which, for some patients (especially those with obesity), may pose a greater challenge.

NW training without dietary intervention in some of the published studies does not affect body weight; thus, the reduction in BM in the SG12 group indicated in this study shows the beneficial effect of combining workouts with TRE. The results indicate that extending the intervention for an additional 12–24 weeks is likely to achieve even better results, but this needs to be confirmed in further studies. It is also worth noting that the proposed interventions led to an increase in TBW in the trained individuals. As early as 6 weeks into the intervention, water content began to increase, and this effect was also observed after 12 weeks of intervention. Although the effect was evaluated to be low, it is still beneficial from a clinical perspective.

In this study, a beneficial trend of decreasing concentrations of TC, TG, and LDL was observed in the fasting group for 6 weeks. In the fasting group for 12 weeks, these concentrations slightly increased. Opposite changes were observed for HDL in both groups. However, these differences were statistically insignificant. Similar favorable changes in the lipid profile after 6 weeks as those obtained in this study in the SG6 group, but statistically significant, were observed in the study by Naseer et al. [50]. This may indicate that a shorter intervention may have a more beneficial effect. In the study by Kortas et al. [13], these parameters were also evaluated in combination with TRE and NW but in an older age group. Similar to our study, no statistically significant differences were obtained. Therefore, it can be suggested that physical activity in groups with a high burden should be used as an adjunctive method in the treatment of dyslipidemia rather than as a form of monotherapy.

It was indicated that TRE may affect blood morphological parameters. In a study conducted on patients with sickle cell anemia, a monthly TRE regimen during Ramadan influenced, in both women and men, a non-significant decrease followed by an increase in leukocyte count [51]. Similarly, in both fasting groups in this study, there was an increase in leukocyte count, although it was statistically insignificant. The cited study also reported a significant increase in platelet count after a 4-week period of TRE, whereas in the groups in this study, the changes were not statistically significant. In the group undergoing fasting with training for 6 weeks, there was a decrease in platelet count, while in the SG12 group, there was a slight increase. However, the magnitude of changes in both groups was not clinically significant.

In the study by Gasmi et al. [52], it was demonstrated that a 12-week fasting regimen can influence changes in blood morphology parameters in both older and younger men. Significant decreases in hematocrit, white-blood-cell count, lymphocytes, and neutrophils were observed in the participants. In our study, a 12-week fasting group also showed a decrease in hematocrit values; however, it was not statistically significant. Meanwhile, in both fasting groups, the leukocyte count increased, which may be related to the training effect, similar to the observed increases in neutrophil and lymphocyte counts. A similar effect was observed in patients with multiple myeloma participating in a 6-week cycle of Nordic walking training, where an increase in leukocyte count was also noted [8].

The combination of dietary intervention and Nordic walking training was also studied in the context of peripheral blood morphology. In the study by Kortas et al. [13], blood morphology parameters were evaluated after 12 weeks of TRE and NW training. Similar changes were observed in the present study regarding the white-blood-cell count, both after 6 and 12 weeks of intervention. Similarly, the number of erythrocytes changed in a similar manner after 6 and 12 weeks, although in this study, these changes were not statistically significant. The increase in leukocyte count may indicate stimulation of the immune system due to training combined with fasting. Training combined with fasting led to a significant decrease in the HCT level in SG12, which could be related to an increase in blood volume. In this context, a slight decrease in the RBC count observed should not be associated with impaired erythropoiesis. Nevertheless, it is worth emphasizing that all observed results fell within the range of the reference values, and further directions of changes associated with longer-term TRE would require additional research.

In this project, changes in the concentration of two selected adipokines under the influence of interventions were also assessed. The mean concentration of leptin in the studied population was 29.0 ± 15.9 ng/mL, which, despite higher values of this adipokine in women than in men [53], still indicates exceeding reference values and informs about pathologically high values. As indicated by previous observations, training durations below 12 weeks do not affect the concentration of this adipokine (except in patients with diabetes) [54]. It was also demonstrated that training has a stronger impact on leptin concentration in women than in men [55]. The results of this study complemented these data by showing that the combined effect of 12 weeks of training and TRE is still not a strong enough stimulus to induce a change in the leptin concentration. However, exercise protocols that result in fat mass reduction (as was the case in this project) lower the leptin concentration. Therefore, most researchers report a decreased leptin concentration after achieving fat tissue loss [54]. However, there are conflicting results regarding long-term (>12 weeks) exercise studies, with many studies showing no effect on leptin concentration. For example, studies by Pasman et al. [56] showed that the exercise-induced decrease in leptin concentration is independent of fat tissue content and serum glucose concentration. Similarly, in this project, no significant correlations were found between the magnitude of differences obtained before and after the training series and body composition (except for a correlation with SLM r = 0.319). However, a relationship between baseline leptin concentration and body composition was indicated: BM, VFA, and the strongest correlation with BF. Negative correlations were observed for SLM. An interesting observation is the indication that the change in leptin concentration was negatively correlated with its baseline concentration, which can be interpreted as indicating that, in individuals with pathologically high leptin concentration (leptin resistance?), changes induced by diet and training will be more challenging to achieve.

The average concentration of resistin in individuals in this project was 17.8 ± 6.4 pg/mL. The resistin concentration in healthy individuals does not differ by age and sex and correlates with BMI [57]. However, in individuals with diabetes, resistin levels are not correlated with BMI. In this project, no correlation was found between resistin concentration and BMI. On the one hand, this may indicate the presence of glycemic disturbances in the studied population or be a consequence of the group selection, where the minimum BMI was 25. Thus, the distribution of this variable did not allow for a correct correlation analysis. Measurements after the specified time (6 or 12 weeks) showed lower resistin concentrations than the baseline results. A high resistin concentration is associated with insulin resistance [57], so it can be indicated that the proposed interventions led to favorable changes in a population characterized by metabolic disorders, including carbohydrate metabolism disorders. However, the results obtained in this project do not unequivocally confirm this hypothesis. 

### Study Limitations

The main limitation of this study was the small number of participants in each group. Future studies should take this fact into account.

## 5. Conclusions

The combination of Nordic walking training and time-restricted eating is a well-tolerated intervention for individuals with an abnormal body composition. Extended to 12 weeks, this intervention allows for improvement in body composition with a noticeable, strong effect on reducing fat tissue content. Neither 6 nor 12 weeks of training and fasting affected the lipoprotein profile, indicating the need for dietary modification not only concerning meal timing but also their quantity and quality. To achieve favorable effects, dietary consultations or pharmacological support are recommended. The conducted interventions did not affect leptin concentration. However, they allowed for a decrease in resistin concentration, which may indicate an improvement in carbohydrate homeostasis in the studied women despite no changes in fasting glucose concentration, although this requires confirmation in further studies. An interesting observation is the indication that the change in leptin concentration was negatively correlated with its baseline concentration, suggesting that, in individuals with a pathologically high leptin concentration, changes induced by diet and training will be more difficult to achieve.

The practical implication is that it should be communicated that interventions that last longer should be used to prepare those undertaking such dietary and training interventions so that their beneficial effect will occur after a longer period of time. In our opinion, such clear communication will contribute to a lower drop-out rate in the absence of effects achieved in short protocols.

## Figures and Tables

**Table 1:** Basic characteristics of the studied women.

Parameter	Total (Baseline)	Group	Before	After	*p*, η^2^
Body Hight [cm]	162.08 ± 5.78	SG6	162.67 ± 5.46		>0.05
		CON6	160.44 ± 4.45		
		SG12	161.01 ± 6.41		
		CON12	164.06 ± 6.38		
Body Mass [kg]	77.39 ± 17.73	SG6	79.57 ± 8.04	77.98 ± 8.22	Time: 0.018, 0.132 Group: 0.053, 0.173 Time × group < 0.001, 0.333
		CON6	88.54 ± 25.42	89.62 ± 25.38	
		SG12	67.79 ± 13.48	66.82 ± 12.79 *	
		CON12	77.25 ± 18.44	78.00 ± 18.92	
Lean Body Mass [kg]	47.39 ± 6.49	SG6	48.96 ± 4.83	48.53 ± 4.37	Time: 0.512 Group: 0.107 Time × group: 0.733
		CON6	50.23 ± 5.98	50.66 ± 5.85	
		SG12	43.78 ± 5.73	44.04 ± 6.05	
		CON12	47.62 ± 8.03	46.94 ± 7.72	
Total Body Water [kg]	34.10 ± 5.08	SG6	35.25 ± 3.49	34.94 ± 3.17	Time: 0.296 Group: 0.163 Time × group: 0.158
		CON6	36.78 ± 5.12	37.06 ± 5.00	
		SG12	32.18 ± 4.21	32.38 ± 4.44	
		CON12	32.93 ± 6.60	34.40 ± 5.59	
Total Body Water [%]	45.23 ± 4.88	SG6	44.33 ± 1.59	44.91 ± 1.98 *	Time: 0.049, 0.096 Group: 0.059, 0.172 Time × group: 0.332
		CON6	42.12 ± 19.88	42.66 ± 15.13	
		SG12	48.37 ± 6.44 #	49.35 ± 6.75 #*	
		CON12	43.18 ± 4.86 #	44.02 ± 4.75	
Body Fat [kg]	30.32 ± 12.92	SG6	30.43 ± 4.25	29.47 ± 4.69	Time: 0.021, 0.126 Group: 0.074, 0.158 Time × group: 0.060, 0.167
		CON6	38.31 ± 20.76	38.99 ± 20.73	
		SG12	24.02 ± 9.92	22.78 ± 9.83	
		CON12	31.10 ± 11.92	31.06 ± 11.83	
Body Fat [%]	37.32 ± 6.39	SG6	38.24 ± 2.47	37.64 ± 2.74	Time: <0.001, 0.784 Group: 0.103 Time × group: 0.143
		CON6	38.74 ± 4.01	39.26 ± 4.51	
		SG12	34.19 ± 8.75	32.87 ± 9.17 *	
		CON12	38.99 ± 6.90	38.58 ± 6.45 *	
Body Mass Index [kg/m^2^]	29.35 ± 6.42	SG6	30.04 ± 2.29	29.46 ± 2.57	Time: 0.158 Group: 0.020, 0.216 Time × group: 0.012, 0.238
		CON6	34.29 ± 10.18 #	34.77 ± 10.05	
		SG12	29.41 ± 4.53	27.95 ± 4.45 *	
		CON12	28.43 ± 5.57	28.89 ± 5.91	
Visceral Fat Area [cm^2^]	139.75 ± 35.10	SG6	136.09 ± 34.68	130.73 ± 37.95	Time: 0.164 Group: 0.692 Time × group: 0.316
		CON6	143.80 ± 45.07	145.20 ± 43.24	
		SG12	134.09 ± 33.43	130.03 ± 35.43	
		CON12	147.47 ± 32.89	149.05 ± 33.95	

SG6—group subjected to training intervention and time-restricted eating for 6 weeks; CON6—control group observed for 6 weeks; SG12—group subjected to training intervention and time-restricted eating for 12 weeks; CON12—control group observed for 12 weeks; *—statistically significant differences between before and after measurements; #—statistically significant differences between groups (if the difference was observed in 2 groups—2 # were put in one cell, if in one group in relation to all others—1 # is marked in one cell).

**Table 2:** Lipid profile and indicators based on lipidogram results.

Parameter	Total (Baseline)	Group	Before	After	*p*, η^2^
Total Cholesterol [mmol/L]	5.175 ± 1.155	SG6	5.590 ± 0.748	5.259 ± 0.882	Time: 0.526 Group: 0.820 Time × group: 0.345
		CON6	5.167 ± 0.828	5.230 ± 0.950	
		SG12	5.029 ± 1.403	5.122 ± 1.381	
		CON12	5.009 ± 1.400	4.965 ± 1.301	
High-Density Lipoprotein [mmol/L]	1.608 ± 0.366	SG6	1.572 ± 0.154	1.681 ± 0.447	Time: 0.660 Group: 0.985 Time × group: 0.057, 0.022
		CON6	1.560 ± 0.331	1.705 ± 0.427	
		SG12	1.643 ± 0.420	1.571 ± 0.384	
		CON12	1.641 ± 0.472	1.584 ± 0.395	
Low-Density Lipoprotein [mmol/L]	2.921 ± 1.012	SG6	3.282 ± 0.789	2.919 ± 0.900	Time: 0.545 Group: 0.914 Time × group: 0.360
		CON6	2.810 ± 0.759	2.878 ± 1.011	
		SG12	2.809 ± 1.244	2.916 ± 1.213	
		CON12	2.851 ± 1.138	2.860 ± 1.101	
Triglycerides [mmol/L]	1.416 ± 0.686	SG6	1.617 ± 0.579	1.450 ± 0.393	Time: 0.770 Group: 0.173 Time × group: 0.228
		CON6	1.756 ± 0.953	1.638 ± 0.744	
		SG12	1.259 ± 0.571	1.384 ± 0.752	
		CON12	1.129 ± 0.462	1.143 ± 0.497	
Glucose [mmol/L]	5.508 ± 1.116	SG6	5.287 ± 0.878	5.110 ± 0.687	Time: 0.617 Group: 0.763 Time × group: 0.347
		CON6	5.305 ± 0.616	5.779 ± 2.077	
		SG12	5.671 ± 1.757	5.568 ± 1.645	
		CON12	5.686 ± 0.806	5.421 ± 0.774	
Atherogenic Index of Plasma	−0.081 ± 0.251	SG6	−0.009 ± 0.157	−0.061 ± 0.139	Time: 0.154 Group: 0.176 Time × group: 0.109
		CON6	0.012 ± 0.237	−0.035 ± 0.158	
		SG12	−0.144 ± 0.277	−0.090 ± 0.275	
		CON12	−0.178 ± 0.279	−0.148 ± 0.240	
Low-Density Lipoprotein–High-Density Lipoprotein ratio	1.711 ± 0.768	SG6	1.771 ± 1.014	1.694 ± 0.864	Time: 0.011, 0.023 Group: 0.574 Time × group: 0.048, 0.034
		CON6	1.575 ± 0.867	1.368 ± 0.975 #	
		SG12	1.772 ± 0.716	1.900 ± 0.685 #	
		CON12	1.732 ± 1.014	1.656 ± 0.699	

SG6—group subjected to training intervention and time-restricted eating for 6 weeks; CON6—control group observed for 6 weeks; SG12—Group subjected to training intervention and time-restricted eating for 12 weeks; CON12—control group observed for 12 weeks; #—statistically significant differences between groups (if the difference was observed in 2 groups—2 # were put in one cell, if in one group in relation to all others—1 # is marked in one cell).

**Table 3:** The blood-count results in project participants.

Parameter	Total (Baseline)	Group	Before	After	*p*, η^2^
Leukocytes [thousand/μL]	6.223 ± 1.611	SG6	6.998 ± 1.715	7.477 ± 1.631 #	Time: 0.030, 0.009 Group: 0.017, 0.209 Time × group: 0.448
		CON6	6.859 ± 1.737	7.456 ± 2.442	
		SG12	5.240 ± 0.951	5.683 ± 0.883 #	
		CON12	6.158 ± 1.591	5.915 ± 1.843	
Erythrocytes [thousand/μL]	4.514 ± 0.291	SG6	4.539 ± 0.250	4.479 ± 0.244	Time: 0.038, 0.007 Group: 0.270 Time × group: 0.496
		CON6	4.582 ± 0.266	4.509 ± 0.268	
		SG12	4.396 ± 0.251	4.292 ± 0.247	
		CON12	4.568 ± 0.363	4.539 ± 0.375	
Hemoglobin [g/dL]	13.677 ± 0.840	SG6	13.780 ± 0.767	13.710 ± 0.741	Time: 0.467 Group: 0.638 Time × group: 0.094
		CON6	13.537 ± 0.597	13.671 ± 0.927	
		SG12	13.592 ± 0.818	13.238 ± 0.788	
		CON12	13.762 ± 1.086	13.842 ± 1.115	
Hematocrit [%]	41.044 ± 2.162	SG6	40.490 ± 1.886	40.160 ± 1.968	Time: 0.042, 0.012 Group: 0.184 Time × group: 0.368
		CON6	40.450 ± 1.072	39.986 ± 1.604	
		SG12	41.092 ± 2.093	39.938 ± 2.281	
		CON12	41.831 ± 2.817	41.983 ± 3.151	
Mean Corpuscular Volume [fl]	91.096 ± 3.950	SG6	89.320 ± 3.066 #	89.750 ± 3.149	Time: 0.500 Group: 0.006, 0.257 Time × group: 0.281
		CON6	88.487 ± 4.496	88.614 ± 3.704	
		SG12	93.562 ± 2.872 #	93.108 ± 2.689	
		CON12	91.738 ± 3.859	92.608 ± 3.574	
Mean Corpuscular Hemoglobin Concentration [g/dL]	33.323 ± 1.136	SG6	34.030 ± 0.941 #	34.160 ± 1.475	Time: 0.079, 0.016 Group: 0.012, 0.187 Time × group: 0.370
		CON6	33.462 ± 1.199	34.157 ± 1.036	
		SG12	33.077 ± 0.958	33.154 ± 0.585	
		CON12	32.885 ± 1.223 #	32.958 ± 0.673	
Platelets [thousand/μL]	258.782 ± 71.160	SG6	268.200 ± 65.252	259.400 ± 60.462	Time: 0.880 Group: 0.256 Time × group: 0.296
		CON6	283.625 ± 69.998	277.714 ± 54.322	
		SG12	220.154 ± 44.151	226.923 ± 55.749	
		CON12	274.154 ± 88.579	260.750 ± 74.496	
Mean Platelet Volume [fl]	10.917 ± 0.915	SG6	10.880 ± 0.854	10.820 ± 0.649	Time: 0.240 Group: 0.891 Time × group: 0.323
		CON6	10.688 ± 1.147	11.000 ± 0.929	
		SG12	11.085 ± 0.912	11.108 ± 0.956	
		CON12	10.923 ± 0.893	10.767 ± 0.936	
Platelet Large Cell Ratio [%]	32.328 ± 7.507	SG6	32.240 ± 7.184	31.460 ± 5.246	Time: 0.196 Group: 0.898 Time × group: 0.658
		CON6	30.712 ± 9.582	33.029 ± 7.966	
		SG12	33.692 ± 7.416	33.685 ± 7.632	
		CON12	32.031 ± 7.184	31.192 ± 7.671	
PlateletCriT [%]	0.284 ± 0.064	SG6	0.300 ± 0.063	0.300 ± 0.045	Time: 0.612 Group: 0.037, 0.179 Time × group: 0.566
		CON6	0.321 ± 0.050	0.306 ± 0.047	
		SG12	0.241 ± 0.035 #	0.247 ± 0.044	
		CON12	0.291 ± 0.077	0.278 ± 0.069	
Neutrophils [thousand/μL]	3.477 ± 1.189	SG6	3.971 ± 1.137	4.473 ± 1.190	Time: 0.043, 0.010 Group: 0.043, 0.166 Time × group: 0.255
		CON6	3.757 ± 1.232	4.540 ± 2.249	
		SG12	2.861 ± 0.920 #	3.028 ± 0.917	
		CON12	3.502 ± 1.299	3.316 ± 1.515	
Lymphocytes [thousand/μL]	2.033 ± 0.577	SG6	2.210 ± 0.722	2.156 ± 0.783	Time: 0.909 Group: 0.331 Time × group: 0.608
		CON6	2.271 ± 0.750	2.091 ± 0.476	
		SG12	1.788 ± 0.410	1.950 ± 0.498	
		CON12	1.981 ± 0.396	1.924 ± 0.302	
Monocytes [thousand/μL]	0.520 ± 0.155	SG6	0.640 ± 0.158 #	0.666 ± 0.161	Time: 0.038, 0.010 Group: 0.004, 0.253 Time × group: 0.263
		CON6	0.551 ± 0.169	0.576 ± 0.168	
		SG12	0.431 ± 0.073	0.508 ± 0.097	
		CON12	0.490 ± 0.149	0.471 ± 0.154	
Eosinophils [thousand/μL]	0.149 ± 0.090	SG6	0.135 ± 0.087	0.141 ± 0.087	Time: 0.562 Group: 0.191 Time × group 0.221
		CON6	0.220 ± 0.109	0.203 ± 0.133	
		SG12	0.124 ± 0.095	0.152 ± 0.077	
		CON12	0.144 ± 0.053	0.162 ± 0.077	
Basophils [thousand/μL]	0.062 ± 0.129	SG6	0.042 ± 0.023	0.041 ± 0.020	Time: 0.598 Group: 0.556 Time × group 0.604
		CON6	0.059 ± 0.029	0.073 ± 0.037	
		SG12	0.102 ± 0.240	0.045 ± 0.021	
		CON12	0.042 ± 0.017	0.043 ± 0.015	

SG6—group subjected to training intervention and time-restricted eating for 6 weeks; CON6—control group observed for 6 weeks; SG12—group subjected to training intervention and time-restricted eating for 12 weeks; CON12—control group observed for 12 weeks; #—statistically significant differences between groups (if the difference was observed in 2 groups—2 # were put in one cell, if in one group in relation to all others—1 # is marked in one cell).

**Table 4:** Correlation analysis between body-composition elements and resistin and leptin levels.

		Pearson’s r	*p*			Pearson’s r	*p*
Resistin [pg/mL] I	Resistin [pg/mL] II	0.808	<0.001	TBW [kg]	TBW [%]	−0.423	0.004
Resistin [pg/mL] II	delta% Resistin	0.426	0.004	TBW [kg]	BF [kg]	0.728	<0.001
BM [kg]	LBM [kg]	0.790	<0.001	TBW [kg]	BFP [%]	0.445	0.002
BM [kg]	SLM [kg]	0.658	<0.001	TBW [kg]	BMI	0.735	<0.001
BM [kg]	TBW [kg]	0.872	<0.001	TBW [kg]	VFA [cm^2^]	0.331	0.028
BM [kg]	TBW [%]	−0.670	<0.001	TBW [%]	BF [kg]	−0.899	<0.001
BM [kg]	BF [kg]	0.951	<0.001	TBW [%]	BFP [%]	−0.855	<0.001
BM [kg]	BFP [%]	0.772	<0.001	TBW [%]	BMI	−0.732	<0.001
BM [kg]	BMI	0.930	<0.001	TBW [%]	VFA [cm^2^]	−0.654	<0.001
BM [kg]	VFA [cm^2^]	0.608	<0.001	BF [kg]	BFP [%]	0.920	<0.001
LBM [kg]	SLM [kg]	0.670	<0.001	BF [kg]	BMI	0.950	<0.001
LBM [kg]	TBW [kg]	0.776	<0.001	BF [kg]	VFA [cm^2^]	0.746	<0.001
LBM [kg]	TBW [%]	−0.457	0.002	BFP [%]	BMI	0.864	<0.001
LBM [kg]	BF [kg]	0.583	<0.001	BFP [%]	VFA [cm^2^]	0.778	<0.001
LBM [kg]	BFP [%]	0.310	0.041	BMI	VFA [cm^2^]	0.711	<0.001
LBM [kg]	BMI	0.610	<0.001	Leptin [ng/mL] I	Leptin [pg/mL] II	0.832	<0.001
SLM [kg]	TBW [kg]	0.685	<0.001	Leptin [ng/mL] I	delta% Leptin	−0.334	0.025
SLM [kg]	TBW [%]	−0.323	0.032	Leptin [ng/mL]	SLM [kg]	−0.424	0.006
SLM [kg]	BF [kg]	0.511	<0.001	Leptin [ng/mL]	VFA [cm^2^]	0.446	0.004
SLM [kg]	BFP [%]	0.327	0.030	Leptin [ng/mL]	BM [kg]	0.363	0.021
SLM [kg]	BMI	0.632	<0.001	Leptin [ng/mL]	BFP [%]	0.510	<0.001
				delta% Leptin	SLM [kg] I	0.319	0.045

BM—body mass, BF—body fat, BFP—body mass percent, SLM—soft lean mass, TBW—total body water, LBM—lean body mass, VFA—visceral fat area, BMI—body mass index. I—before; II—after interventions

## Data Availability

The data presented in this study are available on request from the corresponding author due to the bioethics committee decision.
